# Clinical controversies in endoscopic ultrasound

**DOI:** 10.1093/gastro/got010

**Published:** 2013-04-19

**Authors:** Arvind J. Trindade, Tyler M. Berzin

**Affiliations:** Center for Advanced Endoscopy, Division of Gastroenterology, Beth Israel Deaconess Medical Center, Harvard Medical School, Boston, MA., USA

**Keywords:** Endoscopic ultrasound, controversies, gastric cancer, esophageal cancer, contrast enhanced, fine needle aspiration

## Abstract

The field of diagnostic and therapeutic endoscopic ultrasound (EUS) is growing rapidly. Although EUS has enhanced our ability to diagnose and treat a wide variety of GI conditions, there are many controversial issues regarding the appropriate application of EUS techniques. In this review we discuss five controversial topics in EUS: the utility of EUS in staging of esophageal and gastric cancer; selection of appropriate needle gauge for fine needle aspiration (FNA); use of the stylet in FNA; and the emerging role of contrast agents in endoscopic ultrasound.

## INTRODUCTION

Endoscopic ultrasound (EUS) was first introduced into clinical practice in the 1980s and has rapidly evolved into a reliable technique for diagnosis of lesions of the digestive tract. In its infancy in the 1980s, radial echoendoscopes were used only to image lesions of the GI tract [1–4]. With the subsequent advent of fine needle aspiration (FNA), the curved linear array echoendoscope became an important tool for tissue diagnosis [5–6]. Recently endoscopic ultrasound has been used increasingly for interventional endoscopy. Procedures such as EUS-guided biliary access and drainage, fiducial placement and other EUS-guided therapies (e.g. ethanol ablations of tumors) have been performed [7].

Although EUS has enhanced our ability to diagnosis and treat a wide variety of GI conditions, there are many controversial issues regarding the appropriate application of EUS techniques. The purpose of this review article is to describe some of the more common controversies and the current literature surrounding these controversies to help the clinician performing EUS to better understand these issues and optimize his or her practice. We will discuss each issue under a separate heading.

## STAGING OF ESOPHAGEAL CANCER: IS EUS NECESSARY?

According to the National Cancer Institute, the number of estimated new cases of esophageal cancer in the United States alone in 2012 is 17 460 [8], representing the fastest-growing prevalence of any cancer in the western hemisphere. Prognostic and therapeutic decisions in esophageal cancer hinge on accurate tumor staging [9–10]. One of the most common indications for endoscopic ultrasound is esophageal cancer staging. Prognosis of esophageal cancer is related to tumor depth staging (T stage) and lymph node metastasis [10]. Esophageal cancer limited to only mucosa (T1a) has less than a 5–9% chance of metastasis compared with a 19–44% chance of lymph node metastasis with esophageal cancer invading the submucosa (T1b) (staging per the American Joint Committee on Cancer) [11–13]. As a result, esophagectomy is typically recommended for T1b disease, whereas T1a disease may be treated by endoscopic resection [14]. It is therefore important to differentiate between these two T stages. Radiology imaging with computed tomography and magnetic resonance imaging are unable to differentiate between these two T stages and thus endoscopic ultrasound has been recommended to differentiate between early stage cancers [14].

Recently literature has questioned the accuracy of EUS in staging of early esophageal cancer. Young and colleagues systematically reviewed studies comparing EUS staging to definitive EMR or surgery specimens of superficial esophageal cancer and high grade dysplasia in Barrett’s esophagus [15]. They evaluated 12 studies (individual data from 132 patients). EUS correctly predicted the T stage with only 65% accuracy. They concluded that pre-treatment EUS for HGD or intramucosal esophageal adenocarcinoma is unnecessary and might, in fact, be misleading [15]. Pouw *et al.* also performed a retrospective analysis on patients at the Academic Medical Center in the Netherlands with superficial esophageal cancer, who had an EUS preceding an EMR. Of 131 patients, EUS found possible submucosal invasion (T1b staging) in 26 (20%) [16]. However, in 14 of the 26 patients (54%) there was no submucosal invasion on pathology. Per the authors, EUS did not add any information compared to EMR.

More recently Thoasani *et al.* performed a systematic review and meta-analysis comparing EUS staging to EMR specimens or surgical specimens [14]. The authors took a broader approach than the previously mentioned studies by including esophageal squamous cell carcinoma and including studies in languages other than English. Overall, the study included 1019 patients from 19 studies. Their group found that EUS correctly predicted the T stage in 84% of adenocarcinomas and in 81% of squamous cell carcinomas.

Detection of lymph node involvement in esophageal cancer is also a crucial issue and the accuracy of EUS nodal staging has been compared to a variety of other radiological techniques. A pooled summary by Polkowiski is presented in Table 1 [17]. For regional lymph node metastases, all tests had similar overall diagnostic performance: however, the sensitivity of CT and FDG-PET was significantly lower than that of EUS, whereas the specificity was significantly higher. In addition, a study by Shami *et al.* found that about 20% of patients referred for EMR had lymph node involvement on EUS and that EUS-FNA changed course of management [18].

**Table 1:** Direct comparisons of endoscopic ultrasonography, helical or multi-detector row computed tomography and positron emission tomography in the detection of lymph node metastases from esophageal carcinoma. Data extracted from six studies directly comparing EUS to helical or multi-detector row CT and/or FDG-PET (regional and coeliac lymph nodes combined). (Table used by permission of Elsevier.) Polkowski M. Endoscopic staging of upper intestinal malignancy. *Best Practice & Research Clinical Gastroenterology* 2009;**23**:649–661 [17]

Test	Pooled sensitivity	Pooled specificity	Pooled accuracy
	(95% CI)	(95% CI)	(95% CI)
EUS-FNA	0.81 (0.76–0.85)	0.73 (0.63–0.80)	0.77 (0.72–0.81)
CT Scan	0.54 (0.48–0.61)	0.87 (0.79–0.92)	0.65 (0.60–0.70)
FDG-PET	0.52 (0.44–0.60)	0.82 (0.65–0.92)	0.69 (0.60–0.77)

CI = confidence interval, CT = computed tomography, EUS = endoscopic ultrasonography, FDG-PET = 18F-fluorodeoxyglucose positron emission tomography

Overall, EUS in esophageal cancer staging is still recommended by authorities in the field. Heterogeneity among studies are likely related to operator experience, techniques of EUS employed (linear vs radial vs high-frequency mini-probes) and location of tumors. In our own practice, we use EUS to identify early-stage disease (less than T2) for consideration of EMR. The EMR specimen is then used as the definitive tissue staging. We also use EUS for nodal staging, when cross-sectional imaging has failed to identify distant/metastatic disease.

## GASTRIC CANCER STAGING WITH EUS: DOES IT HELP DETERMINE WHO NEEDS NEOADJUVANT THERAPY AND IS IT BETTER THAN CT IMAGING?

Unfortunately, outside of patients involved in gastric cancer screening programs, the majority of patients diagnosed with gastric cancer have advanced disease at time of presentation and are unable to have a curative resection [19].

The purpose of clinical staging gastric cancer is to determine which patients have locoregional/resectable disease versus systemic involvement. The only accepted criteria for unresectable gastric cancer are the presence of distant metastasis and invasion of major vessels such as the aorta and celiac axis (including hepatic and proximal splenic arteries) [20–21].

CT scan, EUS and MRI are all utilized for gastric cancer staging and the method used is often dependent on local expertise. At present, most experts still recommend EUS as the first line for staging, as this technique historically has had the greatest accuracy for both T and N staging. With advances in cross-sectional imaging resolution and technique, the accuracy of MRI and CT are now comparable to EUS for gastric cancer staging. Studies directly comparing these modalities are scarce [17, 22–24]. A meta-analysis has indirectly compared all three modalities and a summary of these results is listed in Table 2 [17]. Where local EUS availability is limited, CT scanning is a reasonable method of staging and can yield comparable results.

**Table 2:** Indirect comparison of diagnostic performance of endoscopic ultrasonography, multi-detector row computed tomography and magnetic resonance imaging in the assessment of gastric carcinoma T-stage, based on 23 studies of EUS, 6 of CT scan and 3 on MRI. (Table used by permission of Elsevier.) Polkowski M. Endoscopic staging of upper intestinal malignancy. *Best Practice & Research Clinical Gastroenterology* 2009;**23**:649–661 [17]

		Detection of serosal involvement	
Test	T-staging accuracy median (range)	Sensitivity median (range)	Specificity median (range)
EUS	0.83 (0.65–0.92)	0.93 (0.78–1.00)	0.88 (0.68–1.00)
CT	0.83 (0.77–0.89)	0.88 (0.83–1.00)	0.94 (0.80–0.97)
MRI	0.73 (0.71–0.83)	0.92 (0.90–0.93)	0.97 (0.91–1.00)

An important controversy with regard to EUS staging of gastric cancer is that it is considered operator-dependent and under-staging of the primary gastric cancer does occur [25–27]. Over-staging may also occur secondary to inflammation surrounding the tumor. Most troublesome is distinguishing T2 tumors (invading muscularis propria) from T3 tumors (penetrating subserosal connective tissue without invasion of visceral peritoneum or adjacent structures) when applying the American Joint Committee on Cancer (AJCC) staging system used in the western hemisphere and in most Asian countries [25–31]. Errors in determining depth of a lesion will affect surgical resectability criteria and the decision to administer neoaduvant therapy [28]. Neoadjuvant therapy is recommended for patients with T2 disease or above, or if lymph node involvement is suspected. On the other hand, early gastric cancer (T1A disease) is potentially amenable to endoscopic removal [19]. Table 3 lists the diagnostic performance of endoscopic ultrasonography in loco-regional staging of gastric carcinoma based on a meta-analysis of 22 studies [17]. Although the sensitivities are high for all T stages, T2 staging is the least accurate, while T3/T4 EUS staging is more accurate.

**Table 3:** Diagnostic performance of endoscopic ultrasonography in loco-regional staging of gastric carcinoma (based on a meta-analysis of 22 studies (Table used by permission of Elsevier.) Polkowski M. Endoscopic staging of upper intestinal malignancy. *Best Practice & Research Clinical Gastroenterology* 2009;**23**:649–661 [17]

Tumor Stage	Pooled sensitivity (95% CI)	Pooled specificity (95% CI)
T1	0.88 (0.85–0.91)	1.00 (1.00–1.00)
T2	0.82 (0.78–0.86)	0.96 (0.94–0.97)
T3	0.90 (0.87–0.92)	0.95 (0.93–0.96)
T4	0.99 (0.97–1.00)	0.97 (0.96–0.98)

Further concern regarding endosonographic overstaging of T2 cancers was demonstrated in a retrospective study performed by Kutup *et al.* in which EUS and histopathology findings were compared [32]. Their group reported that only 19 of 37 T1/T2N0 cases were correctly classified by EUS and those that were misclassified were over-staged and sent for neoadjuvant treatment. In addition, errors with over-staging nodal involvement occur because EUS characteristics of benign versus malignant nodes may not be reliable [17].

In cases where CT has failed to detect metastatic disease, EUS can sometime provide evidence of more subtle metastatic spread. In particular, malignant ascites, detected by EUS near the left lobe of the liver, can be aspirated and sent to cytology to prove metastasis [32]. In addition, EUS may detect lesions in the left lobe of the liver missed by CT scan in patients previously thought to be resectable. Although EUS is helpful in this regard, it should not be used as the sole diagnostic test to rule out metastatic disease [33].

In summary, for both esophageal and gastric cancer, we generally recommend an ‘outside-in’ approach. Cross-sectional imaging such as CT is a useful first step to rule out distant/metastatic disease and may also be accurate for T and N staging. For patients without metastatic or definitive T4 staging on cross-sectional imaging, EUS can then be recommended for additional T and N information.

## DOES NEEDLE SIZE MATTER IN ENDOSCOPIC ULTRASOUND-FINE NEEDLE ASPIRATION OF SOLID LESIONS?

Successful ultrasound-fine needle aspiration (EUS-FNA) diagnosis of pancreatic disease, including pancreatic cancer, was first reported in 1992 [34]. Since then, the indications for EUS-FNA have rapidly expanded and it is considered a very safe method given its low complication rate of less than 2% [35–38]. EUS-FNA is now routinely used for diagnosis of pancreatic masses, subepithelial gastric tumors, esophageal cancer staging, left lobe liver lesions and even adrenal lesions [39–42]. Choice of needle gauge for fine needle aspiration is a key question facing endosonographers. Currently there are three sizes available: 19-gauge, 22-gauge and 25-gauge. The 19-gauge needle is the largest of the three and thus may have the potential to yield the largest quantity of tissue sample, but this may come with a higher risk of bleeding [43].

Most currently available data compare 22-gauge needles to 25-gauge needles and data are limited on the use of 19-gauge needles. There is good quality evidence from randomized prospective trials to compare the sensitivity/specificity of different FNA needles in the diagnosis of pancreatic masses. We advise caution in applying the conclusions of these studies for other type of lesions, especially subepithelial lesions of the upper GI tract.

The first evidence regarding needle size came out in 2009. In 2009, Siddiqui *et al.* performed a randomized, controlled prospective study on 131 patients undergoing EUS-FNA in 131 patients [43]. Sixty-four patients underwent EUS-FNA with a 22-gauge needle and 67 underwent EUS-FNA with a 25-gauge needle. Cytology was diagnostic in 91.6% of patients and did not differ statistically between the two groups. The two groups did not differ in location of the mass, size of the mass, sex of the patients, use of anticoagulatants/NSAID or diagnosis of the lesion (e.g. adenocarcinoma, neuroendocrine tumor, negative diagnosis). They found that the needle size did not make a difference regarding a diagnosis. The number of passes attempted was the same, with onsite cytology present for all cases. There was no difference in ease of needle passage, need to change scope positions secondary to the needle or needle malfunction.

Also in 2009, Lee *et al.* published a study on 12 patients who underwent EUS-FNA for pancreatic and peri-pancreatic masses [44]. All patients had an FNA with a 25-gauge and a 22-gauge needle. Needle order was selected randomly and two passes were taken with each needle. Samples were read immediately after the FNA by cytopathologists, who were blinded to the needle used. A diagnosis was made in all cases by both sizes of needles. There was no difference in the cellularity of the samples obtained between the needles.

In 2011, Fabbri *et al.* published a similar study than Lee *et al.*, with a total of 50 patients [45]. All patients had pancreatic masses and underwent EUS-FNA with both 25-gauge and 22-gauge needles. The results were similar to Lee *et al.*, showing that both needles statistically had the same diagnostic accuracy, with a trend for the 25-gauge needle to yield a better cytologic diagnosis. Diagnostic accuracy was 94% with the 25-gauge needle and 84 % with the 22-gauge needle.

In 2010, Song *et al.* published a randomized, controlled trial comparing 19-gauge and 22-gauge needles during EUS-FNA in patients with pancreatic or peri-pancreatic masses [46]. A total of 177 patients were enrolled: 60 in the 19-gauge group and 57 in the 22-gauge group. Slides of samples were made by endosonographers and retrospectively reviewed by a cytopathologist, who was blinded to the needle being used. Of note, the endoscopist was not blinded, as the needle design is different between a 19-gauge and 22-gauge. Per study protocol, the groups were allowed to change over to the other needle size if the initial needle yielded a non-diagnostic result or there was a technical failure. The diagnostic accuracy per intention- to-treat analysis was similar (19G: 86.7% vs 22G: 78.9% *P* = 0.268). However when evaluating cases by a per protocol basis the 19-gauge group contained a higher success rate (93.9% versus 78.9%, *P* = 0.006). The specimen cellularity was higher in the 19-gauge group (*P* = 0.033). In addition the 19-gauge needle yielded better results in body/tail lesions than the 22-gauge needle (95% versus 76.7%, *P* = 0.031). Finally technical success, as defined by successful needle passage and specimen adequacy, was higher in the 19-gauge needle group vs the 22-gauge needle group (93.9% versus 78.1%,, *P* = 0.006). There were 12 cases where the 22-gauge needle could not yield sufficient tissue to make a diagnosis. The 19-gauge needle was able to make a diagnosis in 11 of these 12 patients. There were five cases in the 19-gauge needle group in which technical failure was observed, because of mass location in the head of the pancreas. Masses in the head of the pancreas require the echoendoscope to be in the duodenum for sampling and the scope is more angulated, making passing the stiffer 19-gauge needle harder. There were no significant complications in either group. This study concludes that the 19-gauge needle may yield more material and may be the preferred choice for pancreatic lesions in the body/tail of the pancreas, where the echoendoscope position is fairly straight and passage of the 19-gauge needle is feasible.

Limitations to the Song *et al.* study have been expressed in a published editorial [47]. First, there was reliance on gross slide examination made by endosonographers, where most studies have an on-site cytologist. In addition, a mean of less than three passes were made from each needle. Previous studies have advocated at least five passes if no on-site cytologist is available. Finally, in the experienced hands of the endosonographers in this study, there were five failures in patients with pancreatic head masses in the 19-gauge needle group and none in the 22-gauge needle group. Thus less-experienced endosonographers must be cautious with the larger 19-gauge needle, given the technical difficulty of passing this needle in certain positions.

Camellini *et al.* published a 2011 study which randomized 129 patients with various GI lesions to EUS-FNA with either a 25-gauge or 22-gauge needle [48]. Their design was a crossover design similar to Song *et al.* This study included lymph nodes and subepithelial upper GI lesions in addition to pancreatic masses, although the majority of patients (84 of 129) had pancreatic masses. Their results were similar to Siddiqui *et al.* in that there was no difference between the 25-gauge or 22-gauge needles in terms of adequacy of samples or number of passes required. There was an advantage for the 25-gauge needle for pancreatic lesions in the uncinate process as a crossover to the 25-gauge needle was successfully performed in four pancreatic masses in the uncinate process.

A recent meta-analysis was published that included the aforementioned studies, as well as six additional studies that were retrospective or prospective and not randomized [49]. The summary findings suggested that 25-gauge needles confer an advantage in tissue adequacy relative to 22-gauge, with no difference in diagnostic accuracy, number of passes, or complications. They found that there was limited data available regarding 19-gauge needles but the available data did not show evidence of improved outcomes with these devices.

In summary, currently available evidence supports the use of the smaller 25-gauge needle, which yields at least comparable results to the 22-gauge and 19-gauge needles and may be easier to maneuver, especially in locations where an angulated echoendoscope is needed. There are no convincing safety data showing any significant advantage between the three commonly used FNA needle gauges but it seems intuitive that a smaller needle that is easier to maneuver would lead to fewer complications over time. It should also be noted that needle size is only one aspect of successful EUS-FNA. Factors not taken into account in studies are skill of the endoscopist, use of the stylet and degree of needle suction and biopsy technique [50].

## DOES THE STYLET AID OR HINDER EUS ASSISTED FINE NEEDLE ASPIRATION?

Fine needle aspiration during EUS is traditionally performed with a stylet in the needle. Once the needle is inside the target tissue, the stylet is first pushed forward slightly, to expel any needle tract tissue, and then removed from the needle. It is thought the stylet helps prevent contamination of normal gastrointestinal tract cells.

Recently it has come into question whether the stylet is necessary for accurate tissue diagnosis. Rastogi *et al.* performed a randomized, prospective trial on 118 patients who underwent EUS-FNA with a 22-gauge needle [51]. Each patient underwent two FNA passes with a stylet and two passes without. The order of the passes was randomized. The lesions were pancreatic masses (*n* = 61), lymph nodes (*n* = 31), liver lesions (*n* = 6), left adrenal (*n* = 5) and other lesions (*n* = 15). The cytopathologist was blinded to the results. The outcomes measured were degree of cellularity, adequacy, contamination, amount of blood and the diagnostic yield of malignancy. The investigators found no difference in quality of specimen or diagnostic yield with or without a stylet.

Sahai *et al.* performed a similar prospective study to Rastogi *et al.* on 135 lesions with a 22-gauge needle [52]. Fifty-eight percent of the lesions were masses (the majority pancreatic masses) and 42% lymph nodes. The use of the stylet was randomized in a 1:2 ratio. This study showed that the stylet did not increase the yield for malignant cells and was in fact associated with poorer sample quality. The authors concluded that the use of the stylet is questionable and requires further investigation.

Wani *et al.* performed a prospective study with 100 patients who underwent EUS-FNA with either a 25-gauge or 22-gauge needle [53]. The number of passes was predetermined by the type of lesion. The order of the passes, with or without the stylet, was randomized. The same outcomes were measured as Rastogi *et al.* These investigators also confirmed no difference in yield and the study was terminated at interim analysis. The presence of a stylet made no difference overall, nor in *per lesion* analysis, in regards to degree of cellularity, adequacy, contamination, amount of blood and the diagnostic yield of malignancy. Wani *et al.* also performed a retrospective case-controlled study that included 228 lesions [54]. Again, the stylet was not shown to make a difference with regards to the before-mentioned end points.

There is considerable evidence, three prospective randomized trials and one retrospective trial, supporting the notion that the stylet does not aid in the sampling for fine needle aspiration; in fact one study showed it may hinder obtaining quality samples. Although larger trials may be needed prior to a consensus, we conclude that it is reasonable not to use the stylet for EUS-FNA in most circumstances.

## CONTRAST-ENHANCED HARMONIC EUS: A FANCY TOOL OR REAL LIFE APPLICATION?

Contrast-enhanced harmonic EUS (CEH-EUS) has been developed to allow detection of microvascular patterns of lesions that could potentially help differentiate malignant from benign disease. It is important to note that per the European literature, CEH-EUS is not indicated for the detection of lesions, but for characterization of lesions already detected by conventional imaging [55].

In CEH-EUS, an intravenous contrast agent is injected that contains microbubbles. On exposure to the ultrasound pulse, these microbubbles oscillate and the transducer is able to detect signals from the microbubbles in vessels, allowing visualization of the parenchymal microvascularture [55–57]. There are three main patterns of vascular enhancement: ‘no enhancement’, ‘homogeneous enhancement/hyperenhacement’ and ‘heterogeneous enhancement’ [58].

**Figure 1:**
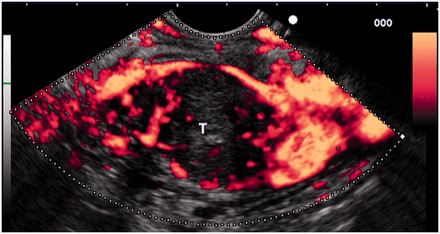
No enhancement of the mass vascularisation with periphericeal hypervascularisation after contrast injection: CE-EUS image of pancreatic adenocarcinoma. (Reproduced by permission of Elsevier.)

One the most useful potential applications for CEH-EUS is in the diagnosis of adenocarcinoma of the pancreas. Contrast enhancement of the pancreas is seen soon after enhancement of the aorta. The enhancement of the mass in question is compared to the surrounding parenchyma. Early studies suggest that, in 90% of adenocarcinoma of the pancreas, the lesion is found to be hypoenhancing on CEH-EUS (Figure 1) [56]. In addition CEH-EUS may help characterize changes in vasculature after chemotherapy, and thus could potentially serve as a useful assessment of response to therapy response to therapy [59].

**Figure 2:**
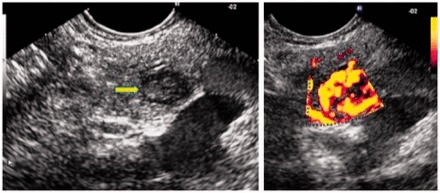
EUS aspects of endocrine tumor of the pancreas. Enhancement of the micro-vascularisation after contrast injection. (Reproduced by permission of Elsevier.)

Perhaps one of the most challenging diagnostic clinical scenarios is differentiating between benign and malignant disease in ‘mass-forming’ acute, chronic and autoimmune pancreatitis (AIP) [60–63]. Mass-forming pancreatitis and autoimmune pancreatitis have similar enhancement to normal pancreas parenchyma on CEH-EUS, where adenocarcinoma is hypoenhancing. These differing imaging characteristics could prove especially useful to help confirm a ‘negative’ FNA during evaluation of a pancreatic mass. CEH-EUS may also distinguish neuroendocrine tumors from adenocarcinoma [56, 64, 65]. Standard EUS can already detect small NET lesions that may not be seen on CT scan. CEH-EUS may provide additional information, given the hypervascular nature of the NET vs adenocarcinoma (Figure 2) [56].

CEH-EUS may also aid in the diagnosis of pancreatic cystic lesions [67–68]. Minute microcystic lesions can sometimes mimic solid lesions on conventional EUS. However, on CEH-EUS, these cystic lesions hyperenhance, given their vascularity. In contrast, pseudo-cysts contain non-vascular debris after their early stages and do not enhance on CEH-EUS.

Finally, an additional potential application for CEH-EUS is in determining the malignant potential of gastrointestinal stromal tumors. 10–30% of GIST lesions are malignant: however preoperative tools to determine malignant potential are not optimal [69]. Obtaining EUS-FNA samples of sufficient cellularity is often challenging. Sakamoto *et al.* conducted a study in 76 consecutive patients who underwent EUS-FNA and CEH-EUS for subepithelial lesions [69]. Twenty-nine patients underwent surgical resection and this group was divided into high versus low malignant potential. The ability of EUS-FNA and CEH-EUS to predict high-grade malignancy in GIST was then compared. CEH-EUS identified irregular vessels and predicted high-grade GIST malignancies with a sensitivity, specificity and accuracy of 100, 63 and 83%, respectively. EUS-FNA had a sensitivity, specificity and accuracy of 63, 92 and 81%, respectively.

## CONCLUSION

The field of diagnostic and therapeutic EUS is rapidly evolving. Recently published work is already helping to clarify some of the important controversies involving the role of EUS in cancer staging and diagnosis, as described in this review. We expect that many of the questions to be debated and clarified in the near future of EUS will pertain to a variety of therapeutic applications of this technique. Of particular interest is the emerging role of EUS-guided biliary access techniques (vs standard percutaneous approaches) for scenarios in which ERCP is not feasible. EUS-guided intra-tumoral therapy (including drug delivery and thermal ablation techniques) and EUS-guided management of variceal bleeding are additional topics of great interest, for which the appropriate role of EUS will need to be thoughtfully investigated.

Along with this growth come controversial issues. In this review we discussed five controversial topics in endoscopic ultrasound. Although the outcomes of endoscopic ultrasound are highly user-dependent and have their limitations, we have shown literature to support its beneficial use in esophageal and gastric cancer staging. In addition, since its first use in the 1980s, we are still learning which techniques are optimal for fine needle aspiration in aiding diagnosis. Two of the common issues regarding needle size and stylet use were presented in this review. More data is probably needed until we have characterized the correct needle size for fine needle aspiration. Factors related to choosing the correct needle correspond to the type and location of the lesion. We briefly discussed the role of the stylet and whether it has a role in fine needle aspiration. Current data suggests that the stylet may not be an essential component of fine needle aspiration. Finally new techniques in EUS are emerging and will likely have a niche in aiding diagnosis of difficult-to-characterize lesions. In this review we have discussed the role of contrast-enhanced harmonic EUS. This technique is evolving and will likely play a key role in diagnostic EUS in the future.

**Conflict of interest:** none declared.
